# Optimization of regeneration and *Agrobacterium*-mediated transformation of Stevia (*Stevia rebaudiana* Bertoni): a commercially important natural sweetener plant

**DOI:** 10.1038/s41598-020-72751-8

**Published:** 2020-10-01

**Authors:** Pooja Taak, Siddharth Tiwari, Bhupendra Koul

**Affiliations:** 1 School of Bioengineering and Biosciences, Lovely Professional University, Jalandhar-Delhi G.T. Road (NH-1), Phagwara, Punjab 144411 India; 2 Department of Biotechnology, Govt. of India, National Agri-Food Biotechnology Institute (NABI), Sector 81, Knowledge City, S.A.S. Nagar, Mohali, Punjab 140306 India

**Keywords:** Biotechnology, Plant sciences

## Abstract

*Stevia rebaudiana* Bertoni is a commercially important zero calorie natural-sweetener herb which produce sweet compounds known as steviol glycosides. Rising demands of steviol glycosides by food and beverage industries has led to an increase in its cultivation in various countries. Unfortunately, stevia cultivation faces 2–25% yield penalty due to weeds which further adds to its cultivation cost. To resolve this major challenge, *Agrobacterium*-mediated genetic transformation of in vitro derived stevia-nodal explants using herbicide resistance gene (*bar*) has been optimized, for the production of stable transgenic stevia plants. Several parameters including explant type, pre-incubation duration, acetosyringone (As) concentration, *Agrobacterium* cell density, Agro-inoculation duration, co-cultivation duration, selection regime and plant growth regulators (PGRs) combination and concentration, have been successfully optimized. Among the two types of explants used, nodal explants showed a higher regeneration response of 82.85%, with an average of 25 shoots/explant. The best PGRs combination and concentration for shoot-induction, shoot-elongation and root-induction was found to be 6-benzyladenine (1.0 mg l^−1^) + naphthalene acetic acid (0.5 mg l^−1^), gibberellic acid (1.0 mg l^−1^), and half-strength MS medium, respectively. The two-step selection (phosphinothricin) regime resulted in an average transformation efficiency of 40.48% with nodal explants. Molecular characterization of putative transformants through PCR, RT-PCR, qRT-PCR and Southern-blot hybridization confirmed the presence, stability, expression as well as copy number of *bar* gene respectively. Compared to the non-transgenic plants, the T_0_ transgenic plants successfully tolerated 8 mg l^−1^ glufosinate ammonium sprays. Thus, the optimized protocol can be useful for the introduction of other genes (inter-kingdom transfer) into stevia genome.

## Introduction

*Stevia rebaudiana* Bertoni (family: Asteraceae) commonly known as ‘honey leaf’, ‘candy leaf’, or ‘sweet herb’ is a perennial shrub of South America.There are 154 species of genus *Stevia*, and among them *S. rebaudiana* is the only species which synthesize steviol glycosides like stevioside, dulcoside A and rebaudioside (rebaudioside A, B, C, D and E) in its leaves. The stevioside and rebaudiosides are 300–400 folds sweeter as compared to cane sugar^1,2^. Although a zero-calorie sweetener, stevia also regulates blood sugar level, body-weight, blood-pressure and prevents tooth-decay^3^. It also possesses anti-oxidant, anti-hyperglycemic, anti-hyperlipidemic, anti-diabetic^4^, anti-microbial, anti-inflammatory, anti-proliferative, anti-cancer and anti-diarrheal properties^5^ etc. According to World Health Organization the daily acceptable dose of steviol glycoside is 0–2 mg kg^−1^of body weight^6^. The increase in the acceptability of this natural sweetener and incidences of diabetes and obesity has led to a large-scale commercial production of stevia crop in developing and developed countries^7^.

The conventional strategies used for stevia cultivation are not reliable because of poor viability of seeds and germination percentage^8^. Hence, to overcome such limitations, in vitro propagation is the only remedy that can facilitate large-scale production of genetically identical stevia plants. Moreover, its cultivation faces several abiotic and biotic constraints. Among the biotic ones, the most important is that stevia is a poor competitor of weeds. The cropping season of stevia is from March to September, and it has to compete with weeds during July to September (rainy season). Several weeds belonging to the plant family Asteraceae, Poaceae, Fabaceae, Solanaceae, Malvaceae, Convolvulaceae, Caryophyllaceae, Brassicaceae, Rubiaceae, Primulaceae, and Plantaginaceae cause reduction in branching and the yield of stevia^9–11^. Therefore, implementation of a robust weed control strategy is crucial for sustainable stevia cultivation.

At present, herbicide applications and mechanical methods are being used abundantly to control excessive weed growth in fields of commercial crops. However, there are smaller number of registered herbicides reported for stevia cultivation which often leads farmers to go for mechanical, hand weeding or certain other chemical practices for weed control. But, chemical methods are more promising and cost-effective as compared to other weed control methods such as mulching (including organic or synthetic mulching), hand weeding and cover crops^12^. Moreover, non-selective herbicides such as paraquat, glufosinate (Basta R) and glyphosate (Roundup R) are toxic for both weeds and crop plants and cause crop injury during field application. Chemical methods include applications of different herbicides also have detrimental effects for crop plant, human being and environment. According to International Agency for Research on Cancer (IARC), two commercial herbicides, glyphosate and 2,4-D are potent human carcinogens^13,14^. Therefore, introduction of foreign gene (for herbicide resistance) into crop plant is highly desirable in order to control weed infestation. The *bar* gene encodes for phosphinothricin acetyltransferase which provide resistance against broad spectrum herbicide phosphinothricin or glufosinate ammonium. Glufosinate is marketed under various brand names (Buster R, Liberty R, Basta R and Finale R) and has some advantages over other herbicides such as, low toxicity and short half-life (low persistence)^15,16^. In this study, *bar* gene has been deployed to develop herbicide tolerant stevia plants.

Several studies have been conducted on in vitro regeneration response of stevia^17,18^ using various explants including nodal sections^19^, leaf^20^, inter-nodal segment^21^, axillary buds^22^, shoot-tip explants suspension cultures, hairy root and anthers^22,23^. However, there are very few reports on optimization of *Agrobacterium*-mediated transformation of stevia.

The major factors which affect the *Agrobacterium*-mediated transformation regime are age and nature of explants, concentrations or combinations of PGRs and physiochemical and temporal conditions of Agro-inoculation and co-cultivation^24^. The objectives of our study were (1) optimization of in vitro regeneration of stevia, (2) optimization of *Agrobacterium*-mediated genetic transformation of stevia with herbicide tolerant *bar* gene, (3) molecular characterization and (4) herbicide tolerance assay of the T_0_ transgenic plants.

## Materials and methods

### Plant material and culture conditions

Three months old stevia plants (variety GVS-16) were obtained from Green Valley Stevia industry, Pojewal, Punjab, India. Leaves, nodal segments and shoot tips from mother plants were taken as explant for in vitro callus regeneration. Explants were surface sterilized with 5% (v/v) Tween 20 for 20 min, washed with tap water, and further treated with 0.1% (w/v) bavistin for 15 min to remove fungal sporesand rinsed 3–4 times with autoclaved Milli-Q water (Merck Millipore, USA). Thereafter, the explants were treated with 0.1% (w/v) HgCl_2_ for 5 min and rinsed 3–4 times with autoclaved Milli-Q water, to remove any traces of HgCl_2_. The explants were then treated with ethanol (70%) for 60 s, followed by several rinses with autoclaved Milli-Q water. The sterilized explants were cultured on MS media^25^ containing 0.3% Phytagel (Sigma, USA), and different combinations and concentrations of PGRs (2,4-D, BAP, KIN, and NAA). Prior to autoclaving (at 121 °C for 15 min), the pH of MS medium was adjusted to 5.7. After autoclaving, the sterile medium was dispensed into sterile jam bottles. All the cultures were incubated at 22 ± 2 °C under white light (PFD: ~ 52 μmol m^−2^ s^−1^) for a photo-period of 16/8 h light/darkness. All experiments were repeated at least three times. Callus formation, shoot regeneration, shoot number and root number, shootlength and root length, were recorded at regular intervals and the cultures were maintained under aseptic conditions.

Rooted plantlets were washed with autoclaved Milli-Q water to remove phytagel media from roots. The plantlets were planted in small plastic pots containing sterile soilrite (soil-conditioning mixture) and regularly irrigated with Hoagland solution. The pots were shielded with transparent perforated polythene sheets to maintain moisture (80–90%) and incubated in a plant growth chamber (Conviron, USA) set at 22 ± 2 °C and 16 light/8 h dark photo-period with PFD of 80 µmol m^–2^ s^–1^. After 22–25 days of acclimatization, polythene bags were removed from the plants.

### *Agrobacterium* strains and vector construct

The plant expression vector pPZP200 *35sde*-*bar*-*loxP* harbouring herbicide tolerant *bar* gene driven by *DECaMv35s* promoter and *loxP* terminator, was used in the study (Fig. 5A). The enzyme phosphinothricin acetyl transferase (PAT) encoded by the *bar* gene inactivates phosphinothricin. The *aadA2* gene encodes an antibiotic (streptomycin-spectinomycin) resistant protein. The construct was used to transform *Agrobacterium tumefaciens* strain GV1301. The active ingredient of the herbicide bialaphos (glufosinate ammonium) was used as selection agent.

### *Agrobacterium*-mediated transformation and explant regeneration

**Figure 1:**
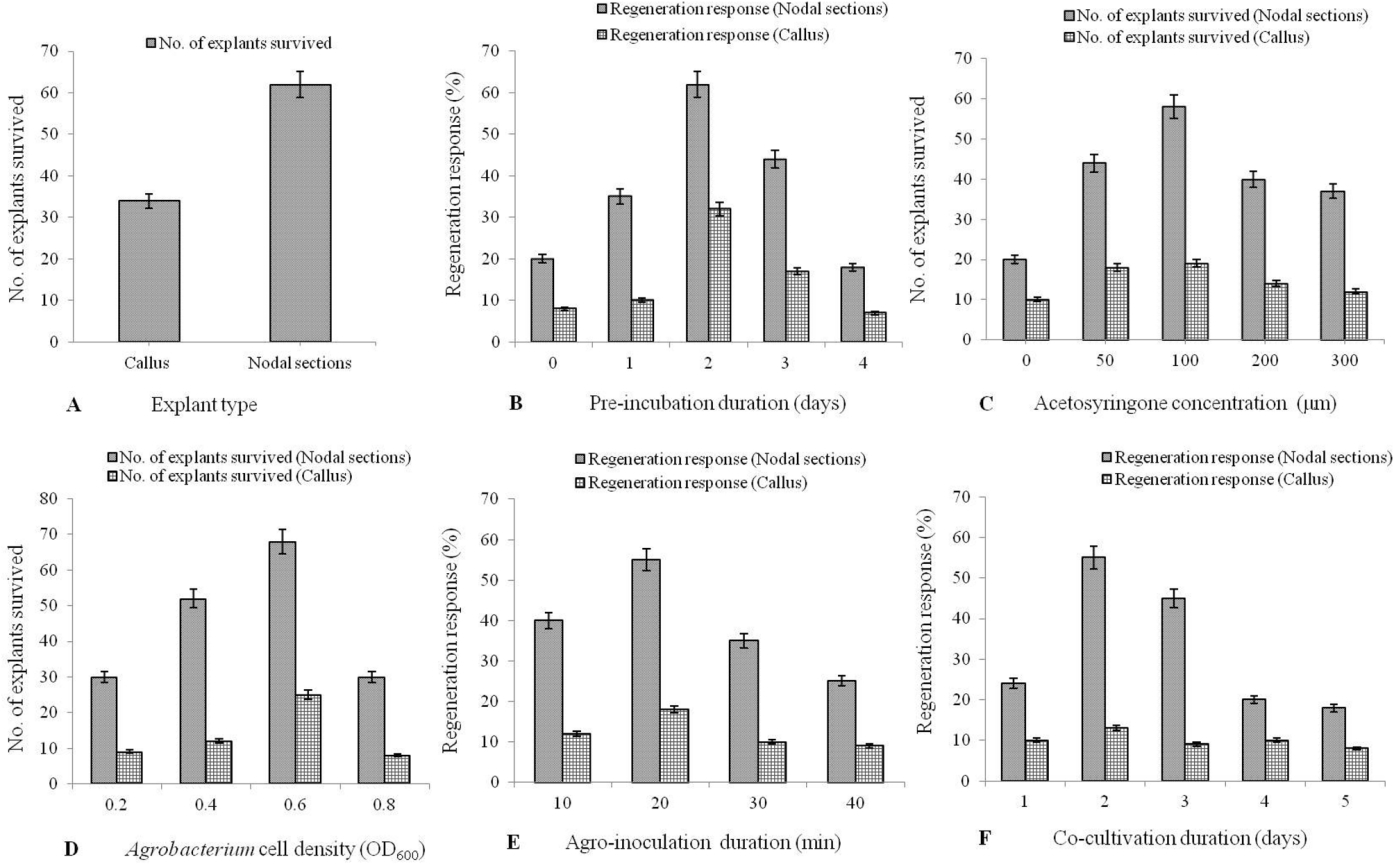
Optimization of *Agrobacterium*-mediated nuclear transformation of stevia. (**A**) Effect of explant type, (**B**) Concentration of acetosyringone (µm), (**C**) O.D_600_ of *Agrobacterium* co-cultivation medium, (**D**) Pre-incubation duration (days), (**E**) Agro-inoculation duration (min) and (**F**) Co-cultivation duration (days).

**Figure 2:**
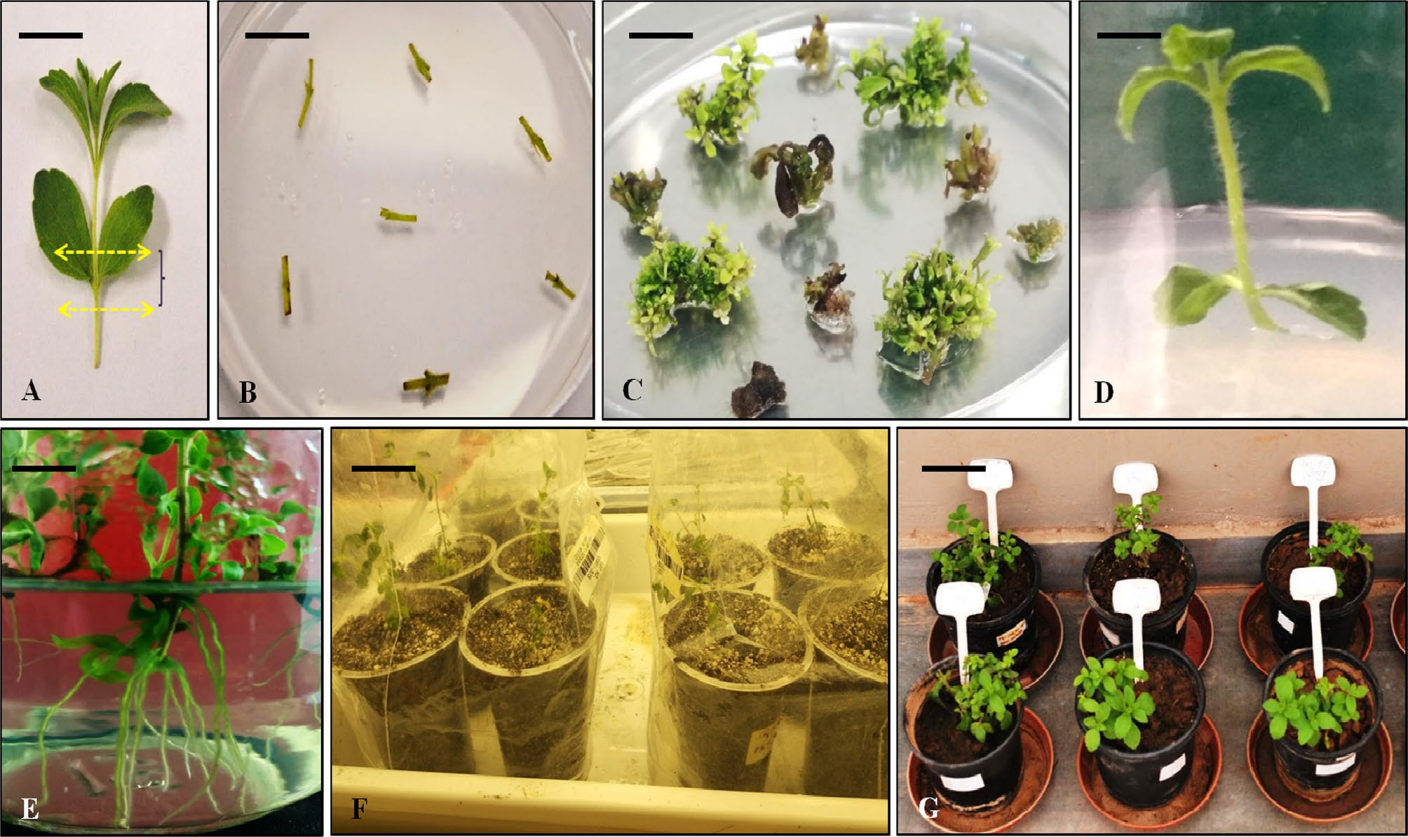
Stevia transformation and *in vitro*regeneration of nodal-explants, (**A**) explant (nodal segments) preparation for agro-inoculation (bar = 1.0 cm), (**B**) explants incubated on co-cultivation media (bar = 1.0 cm), (**C**) regenerated explants subjected to antibiotic selection (bar = 1.8 cm), (**D**) shoot elongation in SEM (bar = 1.8 cm), (**E**) rooting in RIM (bar = 1.0 cm), (**F**) hardening of in vitro raised plantlets (bar = 2.5 cm) and (**G**) green-house grown acclimatized transgenic stevia plants (bar = 4.5 cm).

Nodal sections and leaf originated callus from in vitro regenerated plants (Fig. 2A) were dissected and incubated for 2 days on pre-incubation media containing MS salts + BAP (2 mg l^−1^). These acclimatized explants were used for *Agrobacterium*-mediated transformation with pPZP200-*bar*-*loxP* constructs. Explant wounding was done with sterile needle to enhance the transformation efficiency. Different parameters such as type of explants, pre-incubation duration, acetosyringone (As) concentration, *Agrobacterium* cell density, Agro-inoculation duration and co-cultivation duration were optimized meticulously (Fig. 1A–F). After agroinoculation, the explants were incubated on co-cultivation medium (Fig. 2B) containing MS salts + BAP (2 mg l^−1^) + acetosyringone (As), at 22 °C, in dark for 3 days. Thereafter, the explants were washed twotimes with liquid MS media fortified with cefotaxime (250 mg l^−1^), followed by incubation on MS media containing 250 mg l^−1^ cefotaxime. After 8–10 days, explants were subjected to first selection media (SIM-1) having MS salts + BAP (1 mg l^−1^) + NAA (0.5 mg l^−1^), cefotaxime (250 mg l^−1^) + glufosinate ammonium (2 mg l^−1^) for 22–30 days. The regenerated shoots with a pair of vegetative leaves were identified, excised into segments and were placed on the second selection medium (SIM–2) (Fig. 2C) having the same constituents as SIM–1, except 4 mg l^−1^ of glufosinate ammonium, and incubated for 22–30 days. The independent shoots that regenerated on SIM-2 were transferred to culture-tubes containing elongation medium (SEM) having MS salts + GA_3_ (1 mg l^−1^), and incubated for 15–20 days (Fig. 2D). The elongated shoots were incubated on rooting medium (RIM) containing ½ strength MS medium, for 15–20 days (Fig. 2E). The rooted plantlets were transferred to plastic containers filled with sterile soilrite (soil conditioning mixture) and irrigated with Hoagland solution. The containers were bagged with perforated polythene bags and incubated in a plant growth chamber (Conviron, USA) set at 80% relative humidity, for 15 days of acclimatization (hardening) in soilrite (Fig. 2F). The acclimatized plantlets were planted in plastic pots (12 in.) filled with soil:sand:farmyard manure (3:1:1) and transferred to glasshouse maintained at 24 ± 1 °C under natural light, for normal vegetative and reproductive growth phases (Fig. 2G). The Fig. 4 represents an outline of the optimized protocol for stevia transformation. The percentage transformation efficiency using nodal segments and calluses was determined by the formula as shown below

$${\text{Transformation efficiency}} = \frac{{\text{Independent transgenic events raised after the second selection}}}{{\text{Total number of explants used}}} \times {1}00$$

### Molecular characterization of transgenic plants

Genomic DNA isolation from T_0_ transgenic stevia plants was performed using DNeasy Plant Maxi kit (Cat # 68163, Qiagen, Germany). PCR analysis was done to check the transgene integration and Southern-blot hybridization was performed to check the copy number of transgene^26^. PCR analysis of 100 ng genomic DNA was done by using gene-specific set of internal primers, forward-5ʹCGCCGATGGTTTCTACAAAGA3ʹ and reverse-5ʹTCAATGACCGCTGTTATGCG3ʹ, for the amplification of 146 bp region of *bar* gene with GeneAmp PCR system 9700 (PE Biosystems, USA). PCR amplification cycle used was: initial denaturation: 95 °C (5 min), denaturation: 95 °C (10 s), annealing: 50 °C (30 s), and extension: 72 °C (1 min). For Southern blotting, ~ 10 μg genomic DNA of T_0_ transgenic stevia leaves was digested overnight (12–14 h) with *Eco*RI (Cat #R0101L, New England Biolabs, USA) run on 0.7% gel and transferred on BioBond-plus nylon membrane (Sigma, USA). After pretreatment, the Southern-blot was hybridized for 24 h at 58 °C with α[^32^P] dCTP labeled 552 bp *bar* gene-probe (BRIT, Mumbai, India), followed by three stringent washings. The blots were exposed to Fuji screen for forty eight hours and then analyzed on a phosphoimager (Typhoon Trio^+^, Sweden).

### Reverse transcriptase PCR (RT-PCR) analysis

RNA from leaves of transgenic (T_0_) plants was extracted with TRI reagent (Cat #T9424; Sigma, USA) according to manufacturer’s instructions. For RNA quantification, ND-1000 spectrophotometer (NanoDrop Technologies Inc., USA) was used. DNase (Cat #AMPD1; Sigma, USA) treatment was given to each RNA sample to remove any DNA contamination. cDNA was synthesized from RNA using Power ScriptRT (Cat #RR037B; Takara, Japan) using 5 µg of plant total RNA. Reverse transcription reaction consisted of pre-treatment of 25 μl reaction mixture at 50 °C (10 min), initial denaturationat 95 °C (5 min), denaturation at 95 °C (10 s), followed by and annealing and extension at 60 °C (30 s). Amplification of cDNA fragments was done using specific primers that amplifies 200 bp region of *bar* gene. These amplified gene fragments were then electrophoresed on agarose gel (1%) and analyzed using a gel-documentation system (Bio-Rad, USA).

### Quantitative real-time PCR (qRT-PCR)

SYBR green premix was used to perform qRT-PCR on synthesized cDNA. β-actin gene of Stevia was used as a control (endogenous) for qRT-PCR analysis. Specific primers for β-actin gene were, forward-5ʹTCTTGATCTTGCTGGTCGTG3ʹ, and reverse-5ʹGCGGTTTCAAGTTCTTGCTC3ʹ and *bar* gene specific primers were forward-5ʹGTTTCACCACGTCATCAACG3ʹ and reverse-5ʹTGCCAATTTCCATGTTTGAA3ʹ giving an amplicon size of 150 and 200 bp, respectively. Quantitative PCR was used to analyze the relative quantity of *bar* gene transcript in putative transgenic plants with Quantifast SYBR green PCR kit (Cat. #204054; Qiagen, Germany) using StepOne real-time PCR machine (Applied Biosystems, USA). The reaction mixture (10 μl) comprised of primers (5 pmol), cDNA (1/10-fold diluted), SYBR green (5 μl of 1×) and MQ water (2.5 μl). The experiment was performed using three technical as well as biological replicates.

### Comparison of morphological charactersand chlorophyll content

After shifting the transgenic plants to green-house (Fig. 2G), morphological characters (height of plant, no. of leaves and branches) and chlorophyll content of transgenic and control plants were recorded. For chlorophyll estimation fresh leaves (100 mg) were crushed in acetone (80%), centrifuged at 10,000 rpm for 10 min, at 4 °C. Absorbance of the supernatant was recorded spectrophotometrically (UV-2700 UV–Vis Spectrophotometer, Shimadzu, Japan) at 663 and 645 nm for chlorophyll a and b, respectively. The experiments were performed three times and in triplicates.

### Herbicide tolerance assay

Herbicide tolerance (glufosinate) assay was performed to check the efficacy of transgenic stevia plants for herbicide tolerance as compared to non-transgenic plants. To perform this assay, the wild type (non-transgenic control) stevia plants were divided into five groups, each having five plants (4 test + 1 control). Each group was sprayed (using hand sprayer) with different concentrations of glufosinate (50 ml of 2, 4, 6, and 8 mg l^−1^) under green-house conditions to find the minimum lethal dose for stevia plants. It was observed that 8 mg l^−1^ of glufosinate was the minimum lethal dose for stevia plants. Thereafter, five healthy T_0_ transgenic and non-transgenic control (wild type) stevia plants each were sprayed separately with 8 mg l^−1^ glufosinate in green-house. The experiment was repeated three times and the observations were recorded after 12 days of spray.

### Residual phytotoxic effect

Residual phytotoxic effect of glufosinate on soil was studied by spraying the potted soil with three different concentration of glufosinate i.e. 0.25, 0.50, and 1.0% (v/v) under greenhouse condition. After five days of spray, ten seeds each of indicator plants i.e. corn and cucumber were sown into these pots. The experiment was performed in triplicates and the soil of control plant was sprayed with water as an experimental control. Seed germination percentage was recorded after 10 days of sowing. The plantlet height was recorded with the help of a measuring scale after 20 days of sowing.

### Statistical analysis

Different parameters (percentage of callus formation, number of shoots and roots, were examined using three replicates for each treatment. The values given in tables were expressed as mean ± SD of three replicates i.e. n = 3. Mean and standard deviation were calculated using SPSS software, version 14.0 (SPSS Inc. USA).

## Results and discussion

### Callus induction

In this study, we used different concentrations of 2,4-D (1–3 mg l^−1^), KIN (1–2 mg l^−1^) and BAP (1–3 mg l^−1^) to obtain callus from different explants viz. leaf, nodes and shoot tips of in vitro raised Stevia plants. Callus was initiated from leaf-discs after 4–5 weeks on culture media while, the other explants responded after 6–7 weeks. Hence, leaf discs were most efficient in callus formation and maximum callus induction was achieved on MS2 medium [2,4-D (2 mg l^−1^) and KIN (1 mg l^−1^)] (Supplementary Table 1). Significantly higher callus induction was reported with leaf explants cultured on MS1, MS2 and MS3 media as compared to nodes and shoot tips. The trend observed for callus induction in different media was MS2 > MS3 > MS1 > MS6 > MS5 > MS4 > MS9 > MS7 > MS8.

Leaf discs were found most efficient for callus formation while shoot tips were found least effective. Various researches have been carried out to find the callusing potential of different explants of stevia including leaves^27^, anthers, cell suspension, flower^28^, nodes and roots^27^. Our results are in consonance with that of Janarthanam et al., who found that leaf (juvenile) explants respond better with respect to callus formation (MS media fortified with 2.22 µM BAP and11.31 µM 2, 4‐D) as compared to nodal explants^29^. 80% of callus formation was achieved with leaf segments as compared to 60% with nodal segments. Shooting was achieved from these calluseson MS media supplemented with 1.34 µM NAA and 4.44 µM BAP after 28 days of incubation. In a report by Sairkar et al., leaf discs were found highly efficient for callus formation as compared to nodal segments when incubated on MS media fortified with 1 mg l^−1^ KIN and 2 mg l^−1^ 2,4-D. Subculturing of callus was done after interval of 25–30 days^30^.

### Shoot regeneration

**Figure 3:**
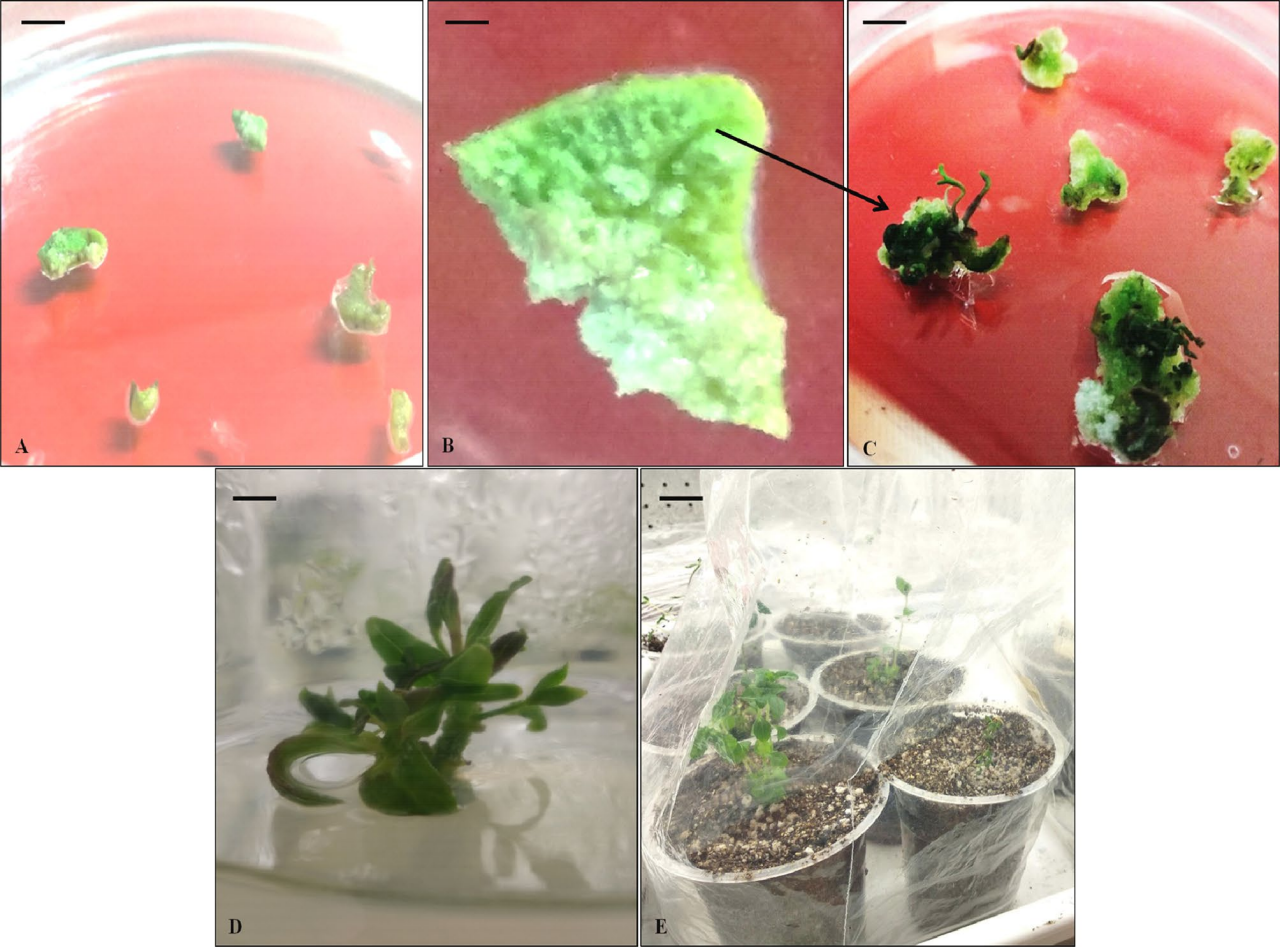
In vitro regeneration of callus explants. (**A**) Leaf discs for callus formation (bar = 1.8 cm), (**B**) callus initiation (bar = 0.25 cm), (**C**) shoot regeneration from callus (bar = 1.8 cm), (**D**) shoot elongation (bar = 2.0 cm) and (**E**) Hardening of in vitro raised plantlets (bar = 4.0 cm).

In this study it has been found that less number of shoots was produced from callus in comparison to direct shoot regeneration from explants (Supplementary Table 2) (Fig. 3). Although, leaf explants derived-callus cultured on MS3 media [BAP (1 mg l^−1^) and NAA (0.5 mg l^−1^)] showed maximum shoot regeneration (4 ± 1.00), the nodal sections cultured on MS6 media [BAP (1 mg l^−1^) and NAA (0.5 mg l^−1^)] exhibited maximum direct shoot regeneration (25 ± 3.2). Direct regeneration of shoots, without any callusing stage is more effective as compared to indirect regeneration. Shoots regenerated through callus are generally asynchronous while directly regenerated shoots are homogeneous, diploid and true-to-type. Significantly higher numbers of shoots were obtained directly from nodal sections cultured on MS4, MS5 and MS6 media as compared to rest of the explants. In a study, a combination of 1.5 mg l^−1^ BAP with 0.5 mg l^−1^ KIN was reported to efficiently induce multiple shoot regeneration from nodal explants^28^. In a study by Debnath, maximum shoot formation was observed with nodal sections and shoot tips cultured on MS media fortified with IAA (1.13 mg l^−1^) and BAP (2.0 mg l^−1^)^31^. In a report by Singh and Dwivedi, they mentioned that the nodal explant showed maximum shoot regeneration (98%) response as compared to shoot tips (55%) and inter-nodal segments (15%). Bud regeneration was reported earlier (in 5.50 days) using nodal section than other explants^32^. Undoubtedly, nodal sections have been the explants of choice for direct shoot regeneration using MS medium fortified with different concentrations of PGRs viz. 1.0 mg l^−1^ BAP and 0.25 mg l^−1^ IAA^33^, 0.5 mg l^−1^ BAP and 2.0 mg l^−1^ KIN^34^. Our report is in agreement with above reports that nodal sections are the choice of explant for efficient and early shoot regeneration.

### Shoot elongation and rooting

The regenerated shoots were cut and further sub-cultured on SEM containing MS media supplemented with various concentrations of GA_3_ (0.5–3.0 mg l^−1^). It has been observed that 1.0 mg l^−1^ of GA_3_ exhibited maximum significant shoot elongation as compared to other concentrations of GA_3_ within 15 days of incubation (Supplementary Fig. 1). According to Sreedhar et al., MS medium fortified with IBA 4.92 µM and 30 g sucrose was most efficient for shoot elongation^35^. GA_3_ was used for shoot elongation in stevia regeneration by Giridhar et al.^36^. It has been reported in their study that 0.05 µM of GA_3_ was most efficient for maximum shoot elongation^36^. Sivaram and Mukundan reported that rooting medium (MS media fortified with 4.90 µM IBA) also acted as shoot elongation medium^37^.

The elongated shoots (~ 2 cm) were transferred to root-induction medium (RIM). In our study, maximum number of roots (9 ± 2.0) was reported from shoots (5–7 cm) regenerated from nodal section, cultured on half-strength MS media devoid of PGR (Supplementary Table 3). No. of roots and root length was found significantly higher in directly regenerated shoots from nodal sections cultured on MS5 and ½ MS media. A comparison between number of shoots and roots originated from directly regenerated shoots and callus regenerated shoots is presented in Supplementary table 2 and 3, respectively. Various research groups have reported root-induction in in vitro regenerated shoots, using different combinations of growth hormones. Singh and Dwivedi reported the maximum rooting response with ¼ MS media augmented with 1.0 mg l^−1^ of IBA + 50 mg l^−1^ of activated charcoal. This media combination produced an average 11 number of roots per shoot^32^. Sreedhar et al. reported best rooting response in shoots incubated on ½ MS fortified with 4.92 μM IBA and 15 g dm^−3^ sucrose^35^.

### Optimization of *Agrobacterium*-mediated transformation

To find the effect of explants type on genetic transformation of stevia, two types of explants (nodal sections and callus) were subjected to Agro-inoculation (O.D_600_ = 0.6). Young nodal sections (0.5 cm) exhibited a high regeneration response of 69.92%, in comparison to a low response of 31.43% with callus explants (Fig. 1A). Different concentrations (50, 100, 200 and 300 µM) of acetosyringone (As) were used to find their effect on transformation efficiency. Supplementation of 100 µm acetosyringone (As) gradually increased the percentage of responding explants to 72.5% while, a lower or a higher concentration (than 100 µM) resulted in reduction of percentage regeneration response (Fig. 1C). Different cell densities (0.2, 0.4, 0.6 and 0.8 O.D_600_ of *Agrobacterium* culture) were used to evaluate their effect on transformation efficiency Maximum regeneration response observed at O.D_600_ was 0.6, while at higher O.D_600,_ *Agrobacterium* contamination was observed on explants (Fig. 1D). Maximum regeneration response (62%) was achieved with a pre-incubation duration of 2 days (Fig. 1B), Agro-inoculation duration of 20 min (55% regeneration response) (Fig. 1E) and co-cultivation duration of 2 days (55% regeneration response) (Fig. 1F). Incubation with *Agrobacterium* enhances the transformation process due to active cell division and formation of *vir*-inducing compounds which enhance the binding of *Agrobacterium* cells on the surface of newly synthesized cell wall^38^.

**Figure 4:**
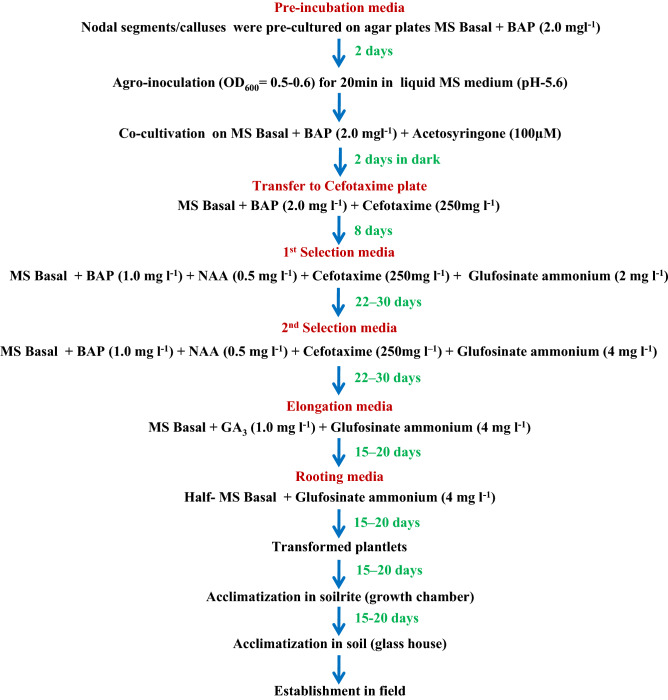
The optimized procedure for *Agrobacterium*-mediated transformation of stevia for efficient recovery of stable transgenic plants.

The optimizations for *Agrobacterium*-mediated transformation and regeneration of stevia are (1) preferred explant type: nodal sections; (2) acetosyringone (As) concentration: 100 μm; preincubation duration: 2 days; (3) *Agrobacterium* cell density (OD_600_): 0.5–0.6; (4) agro-inoculation duration: 20 min; and (5) co-cultivation duration: 2 days; (6) shoot-induction medium (SIM): [MS Basal + BAP (1.0 mg l^−1^) + NAA (0.5 mg l^−1^)]; (7) shoot elongation medium (SEM): [MS Basal + GA3 (1.0 mg l^−1^)]; and (8) root-induction medium (RIM): half-strength MS Basal. After co-cultivation, the explants were cultured on two step selection regime (MS media containing 2 and 4 mg l^−1^ of glufosinate ammonium). A high transformation efficiency of 40.48 ± 0.72% was achieved with nodal sections (Table 1) as compared to 27.94 ± 5.75% with the callus explants (Table 2). The parameters (for shoot regeneration, elongation and rooting) that were optimized for in vitro regeneration of stevia were in consonance with the regeneration after transformation. Figure 4 represent the outline of optimized protocol for stevia transformation using nodal/callus explants.

**Table 1:** Stevia transformation (nodal section explant) and selection on glufosinate supplemented medium.

I selection cycle					II successive selection cycle					
Explant	Number of explants used (A)	Number of responding explants (B)	% response (B/A)	Number of shoots produced	Number of explants used (C)	Number of responding explants (D)	% response (D/C)	Herbicide resistant plants produced	% transformation efficiency	
Nodal sections	80	34	42.00	128	187	87	46.52	35	40.81	40.48 ± 0.72
	80	23	28.75	98	145	112	77.24	44	39.65	
	80	28	35.00	112	143	123	86.01	50	40.98

**Table 2:** Stevia transformation (callus explant) and selection on glufosinate supplemented medium.

I selection cycle					II successive selection cycle					
Explant	Number of explants used (A)	Number of responding explants (B)	% response (B/A)	Number of shoots produced	Number of explants used (C)	Number of responding explants (D)	% response (D/C)	Herbicide resistant plants produced	% transformation efficiency	
Callus	80	15	18.75	50	80	22	27.50	06	27.27	27.94 ± 5.75
	80	10	12.50	58	98	39	39.79	12	31.42	
	80	20	25.00	45	74	20	27.02	04	21.42

### Molecular characterization of putative transformants

**Figure 5:**
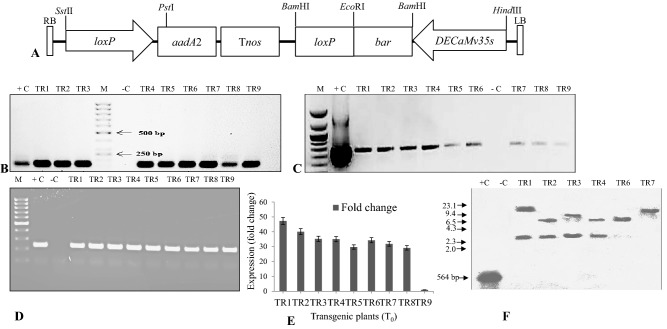
Molecular characterization of T_0_ transgenic stevia plants. (**A**) T-DNA region of pPZP200 vector harbouring *bar* gene driven by *DECaMv35s* promoter, (**B**) PCR amplification of 146 bp of *bar* gene, (**C**) RT-PCR analysis of nine randomly selected transformants showing an amplicon size of 200 bp (*bar* gene), (**D**) RT-PCR analysis of nine randomly selected transformants showing an amplicon size of 150 bp (*actin* gene), (**E**) relative fold change in expression of *bar* gene in T_0_ transgenic plants with respect to TR9 (low expressing transgenic plant taken as reference). *C* control/wild type, *TR* T_0_ transgenic plants, *M* 100 bp ladder and (**F**) southern hybridization analysis of six T_0_ transgenic plants probed with 552 bp *bar* gene. + C: 552 bp *bar* gene (positive control); − C: wild type (negative control).

Genomic DNA and total RNA from randomly selected nine putative transformants (TR1-TR9) were used for *bar* gene integration and expression analyses by PCR, RT-PCR, Southern-blot hybridization and qRT-PCR. PCR result of the nine putative transformants showed amplification of anticipated 146 bp region of *bar* gene which was similar to positive control (plasmid DNA developed with gene-specific primers) (Fig. 5A,B). However, no such amplification was observed with untransformed control plantlets. RT-PCR analysis of the nine promising transformants also revealed amplification of the expected fragment of 200 and 150 bp, which verify the presence of *bar* and *actin* gene transcript in the transgenic plants (Fig. 5C,D). However, the band intensities of the c-DNA amplification product differed in each plant. The TR1 exhibited highest band-intensity while the TR9 exhibited the lowest. The nine T0 transformants were also subjected to qRT-PCR analysis. The *bar* gene expression levels of the nine T_0_ transgenic plants was in consonance with the respective band intensities obtained during RT-PCR analysis. TR9 having the lowest expression level, was taken as reference-control for qRT-PCR result analysis. The fold change in expression of *bar* gene was calculated in terms of 2^−ΔΔCT^ method and plotted on graph^39^. Figure 5E shows ~ 47-fold increase in *bar* gene expression in TR1 transgenic stevia plant as compared to the control (TR9).

Southern hybridization analysis of six highly expressing T_0_ transgenic events revealed the transgene copy number. Genomic DNA from non-transgenic and T_0_ transgenic plants (TR1, TR2, TR3, TR4, TR6 and TR7) was digested with *Eco*RI and subsequently hybridized with 552 bp of *bar*-gene-probe. The hybridization pattern of six T_0_ transgenic plants revealed single and double copy integration that ranged in sizes from 3.5 to 20.5 kb, but the non-transgenic plant (control) did not show hybridization with the gene probe (Fig. 5F).

### Comparison of morphological characters and chlorophyll content

The transgenic plants did not exhibit any significant difference with the control in terms of morphological characters and chlorophyll content (Supplementary Table 4). Average plant height observed in wild type was 73 cm while it was 71 cm in transgenic plants. The leaf count in wild type and transgenic plants was 215 and 211, respectively. Moreover, average chlorophyll content was 7.85 mg g^−1^ and 7.32 mg g^−1^ in wild type and transgenic plants, respectively.

### Herbicide tolerance assay

**Figure 6:**
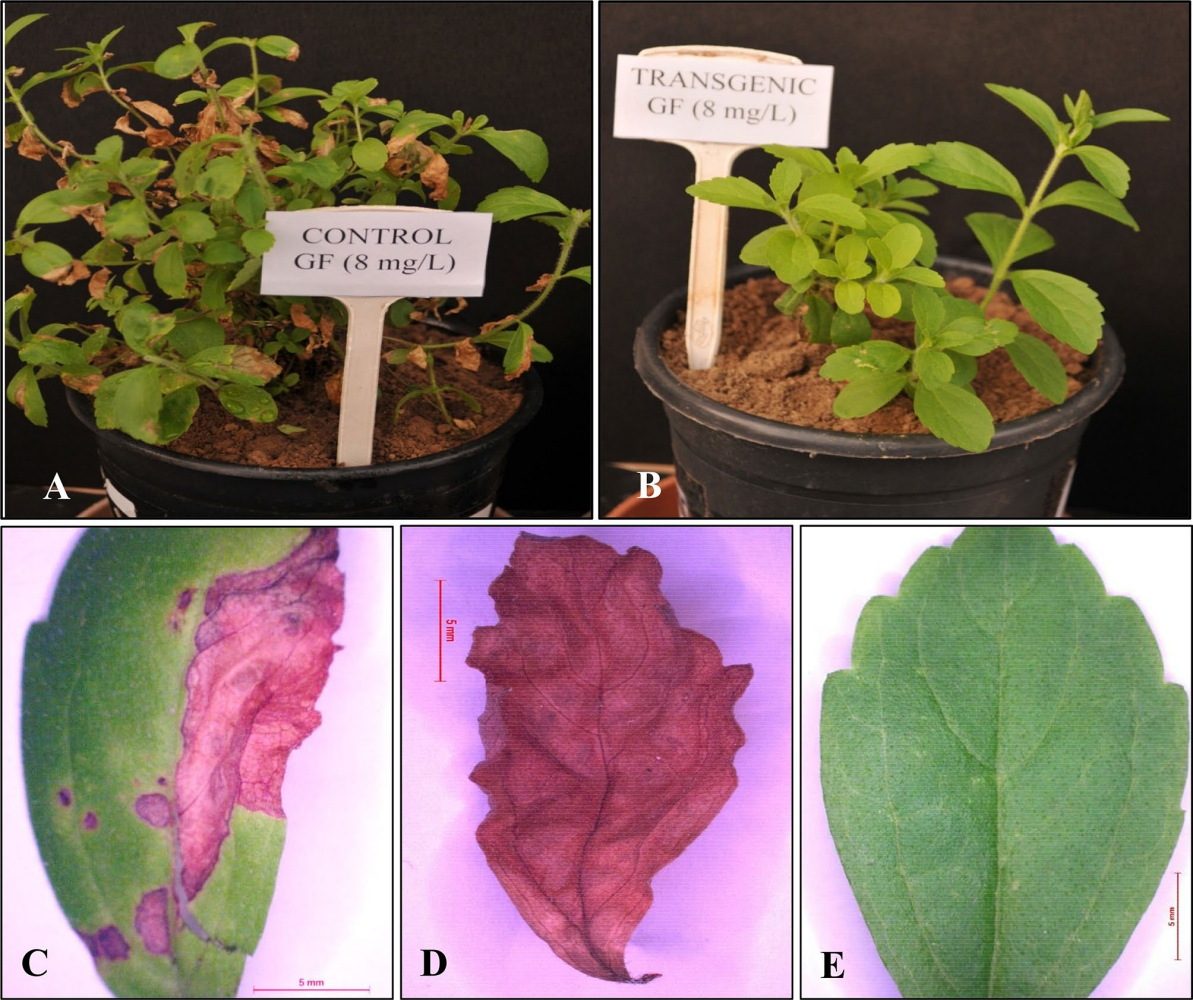
Herbicide tolerance assay of transgenic stevia plants. (**A**) control (wild type) plants sprayed with glufosinate ammonium (8 mg l^−1^), (**B**) T_0_ transgenic plants sprayed with glufosinate ammonium (8 mg l^−1^). Leaf morphology after glufosinate spray (**C**) control leaf after four days of spray, (**D**) control leaf after twelve days of spray and (**E**) transgenic leaf after twelve days of spray. Bar = 5 mm.

Herbicide tolerance assay was performed by spraying the wild type stevia (non-transformed) and T_0_ transgenic plants with 8 mg l^−1^ glufosinate ammonium, in green-house. The wild type stevia plants showed symptoms of chlorosis (Fig. 6A), phytotoxicity and defoliation after 4th day of herbicide spray and even death after 12th day. On the other hand the transgenic plants did not show such symptoms and remained healthy (Fig. 6B–E).

Various reports are available regarding the application of glufosinate on crop plants and weed species. Manickavasagam et al. performed herbicide resistance trials on transgenic (herbicide resistant) sugarcane cultivars Co92061 and Co671^40^. In this study in vitro regenerated non-transformed sugarcane plants were sprayed with different concentrations (0.5, 2.5 and 5.0 g l^−1^) of glufosinate ammonium to find the lethal dose of herbicide. Glufosinate @ 2.5 g l^−1^ with an average dose of 6.25 mg plant^−1^ was observed as lethal dose. This lethal dosage was then sprayed on transgenic plants under greenhouse conditions. Observations were recorded after 30 days to select the transgenic plants. Herbicide resistant sweet potato (*Ipomoea batatas* L.) cultivar “Yulmi” was sprayed and painted with 0.5% glufosinate herbicide (@ 900 mg l^−1^) under greenhouse condition to estimate their efficacy for herbicide resistance. It was found that control plants showed extensive leaf necrosis while transgenic plants remained green without any symptom of leaf necrosis^41^. In a study by Abdeen and Miki, it has been reported that glufosinate spray on Arabidopsis plants led to inhibition of photosynthesis and ultimately plant death, after 6–48 h of spray. While, the transgenic Arabidopsis harboring *bar* gene survived under the experimental conditions^42^. Two Chinese rice cultivars (HD297T-31, HD297T-523) were also transformed with *bar* gene through *Agrobacterium* mediated transformation, for making them resistant to glufosinate herbicide. Transgenic HD297T-31 exhibited almost 100% resistantce to glufosinate while, HD297T-523 showed moderate resistance^43^. In our study, glufosinate adversely affected the wild type (non-transgenic plants), while the transgenic shoots survived on 4 mg l^−1^ glufosinate concentration. Herbicide tolerance assay with T_1_ transgenic jute (*Corchorus* sp.) plants was carried out to analyze their herbicide resistance potential. It was found that control plants died after 12 h of glufosinate spray (0.25%) while transgenic plants successfully recovered from herbicide stress after 7th day of spray^44^. Our results of herbicide tolerance assay are in consonance with these reports.

### Residual phytotoxic effect

No harmful phyto-toxic effect of glufosinate was found on seed germination of both the indicator plants. The difference between parameters (seed germination and seedling length) of control and treated pots was found non-significant. The corn seed germination percentage of 92.78% was observed in water treated pots (control) and 91–92% in glufosinate treated pots. Similarly, with cucumber seed germination percentage of 93.28% was observed in water treated pots and 90–91% in glufosinate treated pots. The corn seedling length of 33.43 cm was recorded in water treated pots and 32–33 cm in glufosinate treated pots. The cucumber seedling length of 7.34 cm was recorded in water treated pots and 6–7 cm in glufosinate treated pots. Moreover, no phytotoxic effect was observed on seedlings of both the indicator plants (Supplementary Table 5).

## Conclusions

Glufosinate (Basta) and glyphosate (Roundup) are two most commonly used broad-spectrum herbicides for weed control in agricultural fields. However, extensive use of a single herbicide induces the weeds to develop resistance against them. At present, nearly 38 weed species has developed resistance to glyphosate, which has been distributed to 37 countries^17^. These resistant weeds are a major challenge to efficient weed control strategies. However, these weeds can be controlled by the strategic application of glufosinate^45^.

In addition to herbicide tolerance, *bar* gene has also been used as a selective marker in many plant genetic transformation experiments^46,47^. In a recent report, cotyledon explants from seven cultivars of soybean were used to introduce *ba*r gene as a selective marker. With a transformation efficiency of 14.71%, transgenic soybean expressing *bar* gene was successfully developed^48^. In our study, much higher transformation efficiency (40.48 ± 0.72) was achieved with nodal sections of stevia plants. This suggests the feasibility of using them for high-frequency *Agrobacterium*-mediated transformation with novel genes of diverse origin.To the best of our knowledge, it is the first report on *Agrobacterium*-mediated nuclear transformation of stevia for herbicide resistance trait. This robust transformation protocol can be useful in stevia crop-improvement programs.

## Supplementary information

Supplementary Figure.

Supplementary Tables.

## Abbreviations

2,4-D: 2,4-Dichlorophenoxyacetic acid
*aadA2* gene: Aminoglycoside nucleotidyltransferase gene
As: Acetosyringone
BAP: 6-Benzylaminopurine
Bar: Bialaphos
*CaMv35sDE*: *CaMv35s* Promoter with duplicated enhancer
GA3: Gibberellic acid
IAA: Indole-3-acetic acid
IBA: Indole-3-butyric acid
KIN: Kinetin
MS: Murashige and Skoog
NAA: α-Napthalene acetic acid
Nos: Nopaline synthase
PAT: Phosphinothricin acetyl transferase
PCR: Polymerase chain reaction
PGR: Plant growth regulator
PFD: Photon flux density
qRT-PCR: Quantitative real-time PCR
RIM: Root induction medium
RT-PCR: Reverse transcriptase PCR
SEM: Shoot elongation medium
SIM: Shoot induction medium
