# Supplementary material for: Optimization of regeneration and Agrobacterium-mediated transformation of Stevia (Stevia rebaudiana Bertoni): a commercially important natural sweetener plant
Source: Sci Rep. 2020 Oct 1;10:16224. doi: 10.1038/s41598-020-72751-8 (PMC7530714; doi:10.1038/s41598-020-72751-8)
Supplement: Supplementary file 1 — Supplementary Figure. [file 41598_2020_72751_MOESM1_ESM.pptx]

## Slide 1
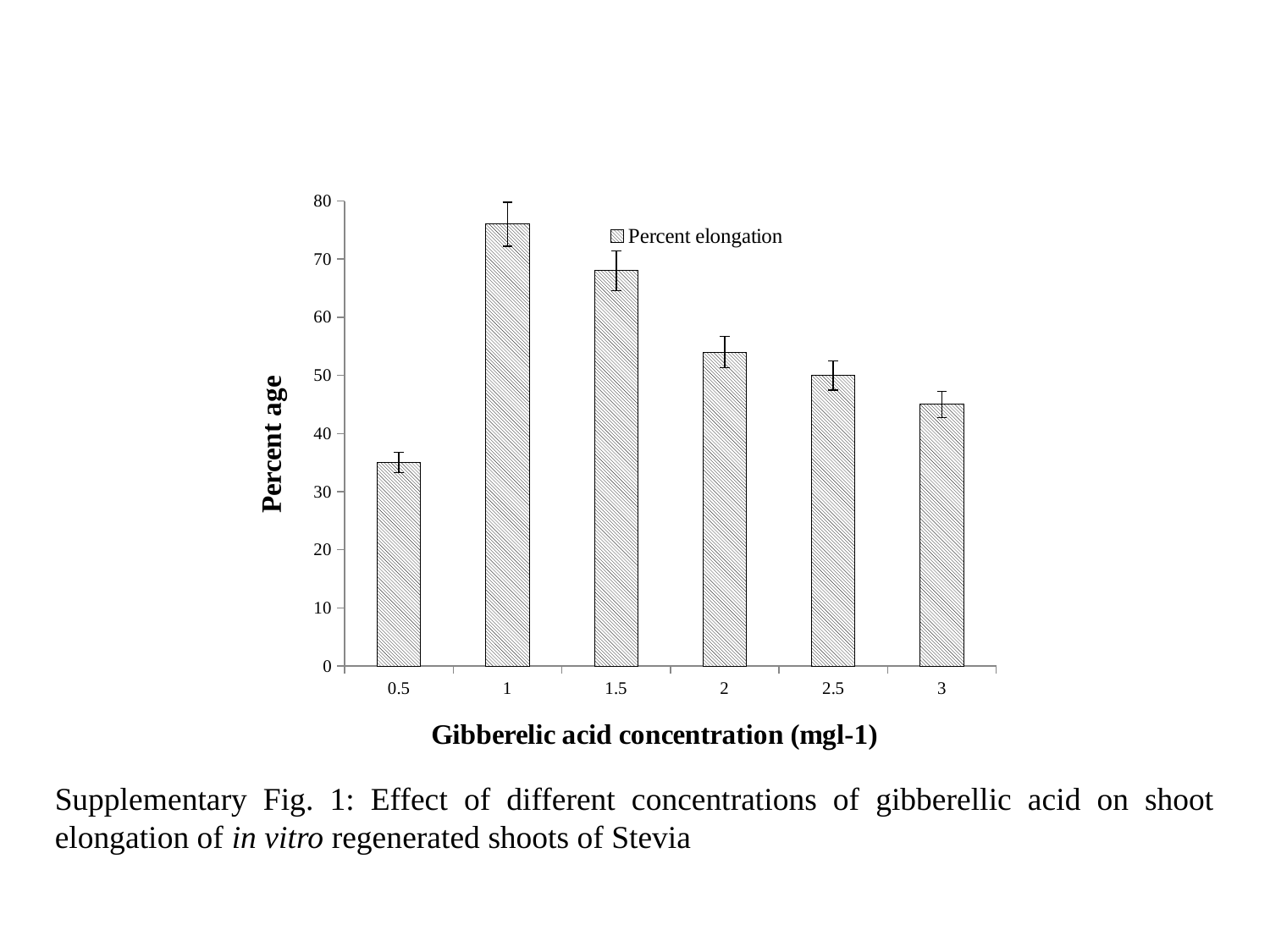

### Chart
| Category | Percent elongation |
|---|---|
| 0.5 | 35.0 |
| 1 | 76.0 |
| 1.5 | 68.0 |
| 2 | 54.0 |
| 2.5 | 50.0 |
| 3 | 45.0 |Supplementary Fig. 1: Effect of different concentrations of gibberellic acid on shoot elongation of in vitro regenerated shoots of Stevia
