# Supplementary material for: Optimization of regeneration and Agrobacterium-mediated transformation of Stevia (Stevia rebaudiana Bertoni): a commercially important natural sweetener plant
Source: Sci Rep. 2020 Oct 1;10:16224. doi: 10.1038/s41598-020-72751-8 (PMC7530714; doi:10.1038/s41598-020-72751-8)
Supplement: Supplementary file 2 — Supplementary Tables. [file 41598_2020_72751_MOESM2_ESM.docx]

**Article type** : Research article

**Title**  : Optimization of regeneration and *Agrobacterium*-mediated transformation of Stevia (*Stevia rebaudiana* Bertoni): A commercially important natural sweetener plant

**Authors**  : Pooja Taak^a^, Siddharth Tiwari^b^*, Bhupendra Koul^a^*

**Corresponding author(s)*** : ^a^School of Bioengineering and Biosciences,

Lovely Professional University,

Phagwara-144411, Punjab, India.

Phone: +91-9454320518

Email: [bhupendra.18673@lpu.co.in](mailto:bhupendra.18673@lpu.co.in)

^b^National Agri-Food Biotechnology Institute (NABI)

Department of Biotechnology, Govt. of India

Sector 81, Knowledge City,

S.A.S. Nagar, Mohali – 140306, Punjab, India

Email: siddharth@nabi.res.in

**Supplementary Table 1** Percentage callus induction using different explants

| **Explant** | **Treatment ID** | **2,4-D (mg/l)** | **Kin (mg/l)** | **BAP**  **(mg/l)** | **Callus induction (%)** |
| --- | --- | --- | --- | --- | --- |
| Leaf | MS1 | 1 | 0 | 0 | 70* ± 5.00 |
|  | MS2 | 2 | 1 | 0 | 82* ± 5.29 |
|  | MS3 | 3 | 2 | 0 | 71* ± 5.56 |
| Node | MS4 | 1 | 0 | 1 | 12 ± 1.73 |
|  | MS5 | 2 | 0 | 2 | 20* ± 1.00 |
|  | MS6 | 3 | 0 | 3 | 24* ± 1.73 |
| Shoot tip | MS7 | 1 | 0 | 0 | 10 ± 0.50 |
|  | MS8 | 2 | 1 | 1 | 9 ± 1.10 |
|  | MS9 | 3 | 2 | 2 | 12 ± 3.60 |

The values are means of three replicates ± SD

**Supplementary Table 2** Direct and indirect *in vitro* regeneration of shoots in stevia

| **Explant** | **Treatments** | **BAP (mg/l)** | **NAA**  **(mg/l)** | **No. of shoots regenerated directly** | **No. of shoots regenerated from callus** |
| --- | --- | --- | --- | --- | --- |
| Leaf | MS1 | 0.1 | 0.0 | 12 ± 1.0 | 1 ± 1.00 |
|  | MS2 | 0.5 | 0.1 | 9 ± 3.40 | 0.00 |
|  | MS3 | 1.0 | 0.5 | 8 ± 1.00 | 4* ± 1.00 |
| Node | MS4 | 0.1 | 0.0 | 18* ± 1.73 | 1 ± 2.00 |
|  | MS5 | 0.5 | 0.1 | 25* ± 5.56 | 0.00 |
|  | MS6 | 1.0 | 0.5 | 25* ± 3.2 | 1 ± 1.00 |
| Shoot tip | MS7 | 0.1 | 0.0 | 5 ± 2.00 | 1 ± 1.00 |
|  | MS8 | 0.5 | 0.1 | 9 ± 2.00 | 0.00 |
|  | MS9 | 1.0 | 0.5 | 13 ± 3.00 | 0.00 |

The values are means of three replicates ± SD

**Supplementary Table 3** Number of roots and root length from callus mediated shoots and directly regenerated shoots

| **Explant** | **Treatments** | **NAA (mg/l)** | **IAA**  **(mg/l)** | **Callus-derived shoots** | | **Directly regenerated shoots** | |
| --- | --- | --- | --- | --- | --- | --- | --- |
|  |  |  |  | **Number of roots per plants** | **Root length (cm)** | **Number of roots per plants** | **Root length (cm)** |
| Leaf | MS1 | 0 | 1 | 1 ± 1.0 | 2 ± 1.0 | 2 ± 1.0 | 4 ± 1.70 |
|  | MS2 | 1 | 2 | 0.00 | 0.00 | 3 ± 2.0 | 3 ± 1.00 |
|  | MS3 | 2 | 0 | 0.00 | 0.00 | 2 ± 1.0 | 4 ± 1.00 |
|  | 1/2 MS | 0 | 0 | 2 ± 1.0 | 2 ± 0.5 | 4 ± 1.1 | 5 ± 1.73 |
| Node | MS4 | 0 | 1 | 3 ± 1.0 | 2 ± 1.0 | 5 ± 1.0 | 4 ± 1.00 |
|  | MS5 | 1 | 2 | 4* ± 1.0 | 4* ± 1.73 | 6 ± 1.0 | 4 ± 2.00 |
|  | MS6 | 2 | 0 | 2 ± 1.0 | 3 ± 1.0 | 7* ± 1.7 | 3 ± 1.00 |
|  | 1/2 MS | 0 | 0 | 7* ± 1.0 | 6* ± 2.0 | 9* ± 2.0 | 7.2* ± 2.90 |
| Shoot tip | MS7 | 0 | 1 | 0.00 | 0.00 | 5 ± 2.0 | 4.2 ± 0.7 |
|  | MS8 | 1 | 2 | 2 ± 1.0 | 2 ± 1.1 | 7 ± 2.0 | 5.4 ± 2.1 |
|  | MS9 | 2 | 0 | 1 ± 0.5 | 2 ± 0.5 | 5 ± 1.0 | 4.4 ± 0.5 |
|  | 1/2 MS | 0 | 0 | 0.00 | 0.00 | 6 ± 1.0 | 6.3 ± 2.4 |

The values are means of three replicates ± SD

**Supplementary Table 4** Comparative analysis of morphological characters and chlorophyll content of control and transgenic plants

| **Parameters** | **Control plants** | **Transgenic plants** |
| --- | --- | --- |
| Plant height (cm) | 73 ± 2 | 71 ± 1.73^ns^ |
| No. of leaves | 215 ± 1 | 211 ± 2^ns^ |
| Total chlorophyll | 7.85 ± 0.23 | 7.32 ± 0.04^ns^ |

ns = Non-significant

**Supplementary Table 5** Effect of Basta herbicide residues (in soil) on germination percentage of indicator plants

| **Basta % (v/v)** | **Germination percentage**  **(10 days after spray)** | | **Plant height (cm)** | |
| --- | --- | --- | --- | --- |
|  | **Corn** | **Cucumber** | **Corn** | **Cucumber** |
| Control (water) | 92.78 ± 0.15 | 93.28 ± 1.03 | 33.43 ± 0.43 | 7.34 ± 0.35 |
| 0.25 | 91.00^ns^ ± 1.23 | 90.17^ns^ ± 0.88 | 32.23^ns^ ± 0.60 | 6.81^ns^ ± 0.71 |
| 0.50 | 92.34^ns^ ± 0.66 | 91.22^ns^ ± 0.28 | 32.00^ns^ ± 0.98 | 7.00^ns^ ± 0.23 |
| 1.00 | 91.87^ns^ ± 1.15 | 90.00^ns^ ± 1.06 | 33.15^ns^ ± 0.43 | 6.73^ns^ ± 0.40 |

The values are means of three replicates ± SD; ns = Non-significant
